# Methylation Status of *H19/IGF2* Differentially Methylated Region in *in vitro* Human Blastocysts Donated by Healthy Couples

**DOI:** 10.6091/.21.1.16

**Published:** 2017-01

**Authors:** Marzieh Derakhshan-Horeh, Farid Abolhassani, Farnoosh Jafarpour, Ashraf Moini, Khadijeh Karbalaie, Sayyed Morteza Hosseini, Somayyeh Ostadhosseini, Mohammad Hossein Nasr-Esfahani

**Affiliations:** 1 Department of Anatomy, School of Medicine, Tehran University of Medical Sciences, Tehran, Iran; 2 Department of Reproductive Biotechnology, Reproductive Biomedicine Research Center, Royan Institute for Biotechnology, Academic Center for Education, Culture and Research (ACECR), Isfahan, Iran; 3 Department of Endocrinology and Female Infertility, Reproductive Biomedicine Research Centre, Royan Institute for Reproductive Biomedicine, ACECR, Tehran, Iran; 4 Department of Obstetrics and Gynecology, Faculty of Medicine, Tehran University of Medical Sciences, Tehran, Iran; 5 Department of Cellular Biotechnology, Cell Science Research Center, Royan Institute for Biotechnology, ACECR, Isfahan, Iran; 6 Isfahan Fertility and Infertility Center, Isfahan, Iran

**Keywords:** Blastocyst, Genomic imprinting, *H19/IGF2* DMR, Human, Reproductive technique

## Abstract

**Background:**

Imprinted genes are a unique subset of few genes that have been differentially methylated region (DMR) in a parental origin-dependent manner during gametogenesis, and these genes are highly protected during pre-implantation epigenetic reprogramming. Several studies have shown that the particular vulnerability of imprinting genes during suboptimal pre- and peri-conception micro-environments often is occurred by assisted reproduction techniques (ART). This study investigated the methylation status of *H19/IGF2* DMR at high-quality expanding/expanded human blastocysts donated by healthy individuals to evaluate the risks linked to ART.

**Method:**

Methylation levels of *H19*/*IGF2* DMR were analyzed by bisulfite conversion and sequencing at 18 CpG sites (CpGs) located in this region.

**Result:**

The overall percentage of methylated CpGs and the proportion of hyper-methylated clones of *H19/IGF2* DMR in analyzed blastocysts were 37.85±4.87% and 43.75±5.1%, respectively. For validation of our technique, the corresponding methylation levels of peripheral human lymphocytes were defined (49.52±1.86% and 50%, respectively).

**Conclusion:**

Considering the absence of *in vivo*- produced human embryos, it is not possible to conclude that the methylation found in *H19*/*IGF2* DMR is actually normal or abnormal. Regarding the possible risks associated with ART, the procedures should be optimized in order to at least reduce some of the epigenetic risks.

## INTRODUCTION

Methylation of cytosine in the 5′ position in CpG dinucleotides, i.e. DNA methylation, is a crucial epigenetic control mechanism in mammals. The most dramatic changes in DNA methylation occur during gametogenesis and early embryo development. For example, the overall DNA methylation of CpG islands in mouse sperm is in the range of 80-90%, which is higher than any other cell in this organism. The maternal genome, however, contains much lower level of DNA methylation (≈40%). Accordingly, paternal genome is actively and rapidly demethylated through the oxidation of 5-methylcytosine to 5-hydroxymethylcytosine by ten-eleven translocation family of enzyme, while the maternal genome is demethylated in a replication-dependent manner[1].

In spite of the global DNA demethylation following fertilization, there is a unique subset of genes, approximately 150 genes in human and mouse[^2^], which are specifically protected from DNA demethylation[3-5]. This class of genes is associated with the genomic differentially methylated regions (DMRs) in a parental origin-dependent manner during gametogenesis. It has been well established that the parent-of-origin specific DNA methylation, known as genomic imprinting, is crucially important for normal embryonic development. Therefore, any interference with imprint acquisition and/or maintenance will result in ill or fatal phenotypes of the resultant embryos[6].

**Fig. 1:**
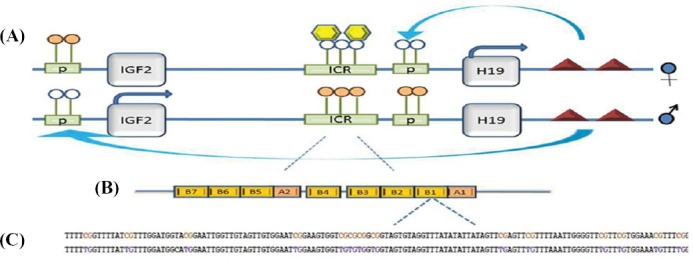
Diagram of *H19/IGF1* imperinted domain regulated by ICR1. A). Diagram of *H19/IGF2* imprinted domain regulated by ICR1 (green rectangles), down-stream enhancers (red triangles), and CTCF (yellow hexagons). Differential methylation of ICR mediates with differential binding of CTCF results in the differential expression of H19 and IGF2; B) ICR1 is formed of tandem repeat elements (B-type repeats, orange boxes) containing CTCF-binding motifs; C) Part of sequence context of B1 repeat assessed in our study [18 CpG sites in a 220-bp fragment of ICR1 (AF125183: 7877–8096)] on paternal (methylated) and maternal (unmethylated) allele in lymphocyte. Unmethylated cytosine in CpGs on maternal allele is converted to thymine (violet color). Methylated cytosine in CpGs on maternal allele remains unchanged (orange CpGs).

In human, most of the imprinted genes are arranged in clusters[7]. As an example, several clusters are located on chromosome 11p15.5 containing two important imprinted genes, *IGF2* and *H19*. The DMR of *H19/IGF2* constitutes an imprint control region (ICR) that regulates nearby imprinted genes. ICR1/*H19* DMR is located between *IGF2* and *H19* in order to regulate the *IGF2* and *H19* expression in a reciprocal manner. ICR1 prevents the activation of *IGF2* promoters through interaction with the zinc finger protein CCCTC-binding factor (CTCF)[8-10]. Therefore, when paternally inherited, DNA methylation inhibits CTCF binding, and ICR1 activity is blocked; hence, *IGF2* is expressed[11]. However, when maternally inherited, ICR1 remains methylation-free, and *H19* is expressed (Fig. 1).

Russell-Silver syndrome (RSS) is a syndrome with pre- and post-natal growth retardation, and Beckwith–Wiedemann syndrome (BWS) is characterized by prenatal and postnatal overgrowth[12]. Hypomethylation of ICR1 is observed in RSS patients[13,14], while hypermethylation of ICR1, which is associated with increased IGF2 expression, is observed in BWS patients[15]. It has been shown that paternally expressed genes (such as *IGF2*) tend to increase fetal growth, whereas those expressed maternally, such as *H19*, restrict fetal growth[16-18].

Although assisted reproductive techniques (ARTs) are now well-established and globally applied, several epidemiological studies have shown the association of ART with the increased incidence rate of certain imprinting disorders such as BWS and RSS[19]. This phenomenon has been mainly attributed to the coincidence between gamete and embryo *in vitro* manipulations events and the normal pattern of epigenetic reprogramming, which begins at fertilization and continues during pre-implantation embryo development[6,20,21].

Despite several investigations in the gametic DMRs (gDMRs) in human pre-implantation embryos, there is still space for further studies. The reason is that in most studies, embryos used were derived from leftover of ART cases and may do not have the best quality or may have been derived from infertile individuals. Considering the possible consequences of reprogramming errors, it is essential to study the normal gDMR DNA methylation status of human blastocyst derived from healthy couples. Therefore, in this study, we determined the methylation status of ICR1 in high-quality blastocysts donated by couples who desire family balancing with proven fertility.

## MATERIALS AND METHODS

### Informed consents and ethical approval

This study was approved by the Ethics Committee of Tehran University of Medical Sciences (no. 92-03-30-24088, 2014/6/7) and Ethical Committee of Royan Institute (Tehran, Iran). All embryos were collected from patients referring to the Isfahan Infertility Center (Isfahan, Iran), and a written informed consent was obtained.

### Source of human blastocyst

Human blastocysts were donated from patients with supernumerary blastocysts referring to the Isfahan Infertility Center for family balancing. A total of 20 expanded or expanding blastocysts were obtained from couples with at least two children of the same sex. Antagonist protocol (Cetroide, Serono) in combination with SinalF (SinaClon, Tehran, Iran) and Menogon (Ferring, Germany) was used for ovulation induction. Ovulation was induced with 10,000 IU of human chronic gonadotropin (IBSA, Switzerland) when three dominant follicles greater than 17 mm were observed in vaginal ultrasound scan. Intra cytoplasmic sperm injection and pre-implantation genetic diagnosis were carried out based on the standard protocols[22], and G5 series sequential media (Vitrolife, Gothenburg, Sweden) was used for all the procedures. Blastocysts were scored according to Gardner *et al*.[22] grading system. After transferring the fresh embryos and cryopreservation of the embryos, the remaining blastocysts were used for this study after receiving a signed informed consent forms from the patients. The embryos of the patients who were not agreed to donation were discarded. The pre-implantation genetic diagnosis results were not revealed to the research group because: i) embryos were scarce, ii) in general ART practices, information on chromosomal status of embryos is not available, and iii) mosaicism is a typical feature of ART embryos[23]. For epigenetic analysis, hatched blastocysts were used. Hatching was induced with the aid of a pipette or removal of zona pellucida with the aid of Tyrode’s acid. Zona-free blastocysts from each couple were pooled and stored in 0.2-ml microtubes at -80°C. Care was taken to make sure there is no granulosa contamination.

### DNA methylation analysis

The DNA methylation of *H19*/*IGF2* DMR was determined by bisulfite conversion and sequencing as described by Borghol *et al*.[24], on a total of 20 blastocysts donated from 6 couple and one million peripheral blood lymphocytes derived from 5 volunteers. Lymphocyte was used as control to obtain the correct pattern of imprinted gene methylation. The genomic DNA from lymphocytes was extracted by salting-out method[25]. The pools of small number of blastocysts (2-3/pool) from the same patient were used per replicate. Blastocysts were thawed and directly placed in a lysis solution (50 mM EDTA, 50 mM Tris, pH 8, 3 µg of RNA carrier, and 0.14 µg/µl proteinase K) in a final volume of 40 µl and then incubated at 55°C for 2 h. Complete bisulfite conversion and DNA clean-up were performed by the EpiTect^®^ Bisulfite Kit (Qiagen, Germany) based on the manufacturer’s. After treatment with bisulfite and purification, DNA was immediately used for nested PCR.

We examined 18 CpG sites in a 220-bp fragment of *H19* (AF125183: 7877–8096) containing the CTCF binding site. Primers specific for bisulfite-converted DNA were as follows: external forward: 5’-GAGTTYGGGGGTTTTTGTATAGTAT-3’; external reverse: 5’-CTTAAATCCCAAACCATAACACTA-3’; internal forward: 5’-TATATGGGTATTTTTGGAGGT TTTT-3’; internal reverse: 5’-ATAAATATCCTATTC CCAAATAACCCC-3’.

The PCR master mix was prepared according to Herman *et al*.[26] with some modifications. Briefly, 30 pmol (300 ng) of each forward and reverse primer was used for PCR reactions with 1× ammonium sulfate buffer (CinnaGen, Iran, CG8108C), 6 mM MgCl_2_ (CinnaGen, TP7506C), 1.25 mM dNTP (CinnaGen, DN7603C), 0.6 U SmarTaq (CinnaGen, TA8108C) and 10 mM 2-mercaptoethanol (Sigma, M7522, USA) in a 50-µl reaction volume. PCR reaction was carried out in an Eppendorf gradient thermal cycler with the following program: 95°C for 10 min, followed by 39 cycles at 95°C for 30 s, 60°C for 30 s, and 72°C for 1 min with a final extension at 72°C for 10 min. The first-round PCR product (2 µL) was used as DNA input for amplification in the second-round nested PCR mix with the following conditions: 95°C for 10 min, 39 cycles at 95°C for 30 s, 60°C for 30 s, and 72°C for 1 min, followed by a final extension at 72°C for 20 min. Four independent nested PCRs were performed per template to avoid amplification bias, which may lead to preferential amplification of maternal or paternal DMRs[21]. PCR products were sub-cloned into a pTZ57R/T cloning vector (InsTAcloneTM PCR Cloning Kit, Fermentas, Lithuania) according to the manufacturer’s protocol. Ligated vectors were transferred into the DH5α strain of *E. coli*, and grown colonies were selected by PCR analysis through the M13 primers. Plasmids from positive colonies were then extracted by the Qiaprep® Spin Miniprep Kit (Qiagen, Germany) and sequenced using standard M13 primers. Clones were also sequenced for each independent nested PCR product. A total of 50 negative PCR controls were tested to verify there is no PCR contamination.

The obtained sequences were analyzed using bisulfite sequencing DNA methylation analysis software. The lower threshold conversion rate and sequence identity were chosen at 90%. The upper threshold of N-sites at cytosine positions and insertions/deletions were set at 20%.

**Fig. 2:**
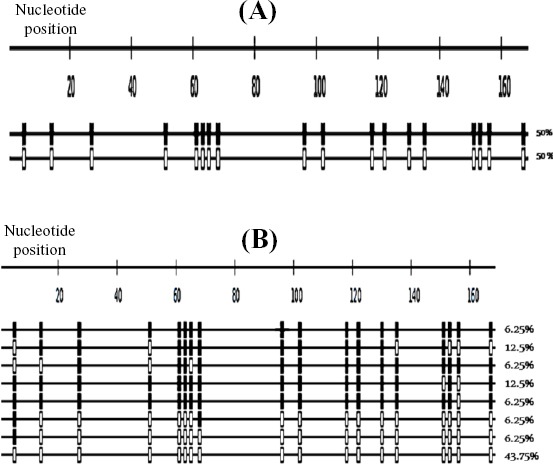
Bisulfite sequencing analysis of *H19/IGF2* DMR. The sequencing result of *H19* DMR in a representative replicate from the A) lymphocyte and B) the blastocyst. Each row indicates a unique DNA clone. Filled and open squire represent methylated and unmethylated CpGs, respectively. The number of clones is presented as percentage (%). In these replicates, the total number of clones is 16.

Final analysis of the included sequences are presented in Figure 2, where the clones by default are sorted according to their methylation level. The Figure shows the methylated and unmethylated cytosines observed in each sequence at the reference CpG sites in context of the sequence length. Bisulfite sequencing DNA methylation analysis presents a methylation statistic for the overall methylation and the average methylation for each clone. Furthermore, the calculated average methylation at each CpG position was presented.

The methylation status of the H19 DMR was determined by cloning and sequencing of bisulfite-treated DNA. To reduce amplification bias of maternally- or paternally-derived genome copies, the pools of a small number of blastocysts (2-3/pool) from the same patient were analyzed. In addition, more than 15 clones were sequenced per replicate for four independent PCR products. The efficiency of bisulfite PCR amplification from blastocysts was 66.6%.

### Statistical analysis

The proportions of hyper-methylated clones and the percentages of overall methylation were determined to be normally distributed, with the homogeneity of variances (Leven’s test). To assure the accuracy of the results, the experiment was repeated at least four times.

## RESULTS

### Methylation status of the *H19/IGF2* DMR

Our bisulfite sequencing protocol was validated on genomic DNA extracted from human peripheral blood after several independent experiments, indicating no bias among methylated or unmethylated alleles. Therefore, the methylation status of lymphocyte was used as a template for validation of methylation status of the embryos (Fig.2A)[24,27].

### Percentages of overall methylated CpGs at *H19/IGF2* DMR

To identify DNA methylation patterns of *H19/IGF2* DMR, the region including 18 CpGs was analyzed. The percentage of overall methylated CpGs of the blastocysts in the region was 37.85±4.87%. The detail of methylation status in ICR1 sequence in a representative replicate is presented in Figure 2B, Tables 1 and 2. Furthermore, the methylation pattern of lymphocyte was 49.52±1.86% (Fig. 2A).

**Table 1:** The number of CpG sites, CpG position and percentage of methylation for each CpG site in ICR1 sequence in a representative replicate in the blastocyst

CpG site	CpG position	Methylation (%)
1	5	37.50
2	14	37.50
3	27	43.70
4	15	25.00
5	61	43.70
6	63	43.70
7	65	37.50
8	68	50.00
9	96	43.70
10	102	43.70
11	118	43.70
12	122	43.70
13	130	43.70
14	135	31.25
15	151	31.25
16	153	31.25
17	156	12.50
18	167	31.25

**Table 2:** DNA methylation summary overall sequences in a representative replicate located in ICR1 sequence in the blastocyst

DNA methylation summary overall sequences	The number of CpG (180 cases of 288) (%)
Unmethylated CpGs	62.5
Methylated CpGs	37.5

### Percentages of hyper-methylated clones of *H19/IGF2* DMR

The clones with >50% of the CpGs methylated are considered as hyper-methylated, and strands lacked nine or more methylated CpGs were considered to be hypo-methylated[28-30]. Using these gauges, the percentage of hyper-methylated clones of *H19*/*IGF2* DMR in the blastocysts was 43.75±5.1%, while this value for the lymphocyte was 50% (Fig. 2, Table 3).

**Table 3:** Average methylation for each clone in a representative replicate located in ICR1 sequence in the blastocyst.

Seq. identifier	Average (%)
1	100
2	66.6
3	66.6
4	94.4
5	88.8
6	88.8
7	77.7
8	0.0
9	0.0
10	0.0
11	0.0
12	0.0
13	0.0
14	0.0
15	5.5
16	11.1

The number of clones is shown in the raw of “Seq. identifier”. Seq, sequence

## DISCUSSION

Thirty six years after the birth of first child through *in vitro* fertilization and with the advent of new techniques, including cryo-preservation, intra cytoplasmic sperm injection, pre-implantation genetic diagnosis, and assisted oocyte activation, ART is now accounting for a considerable proportion of child birth. In some European countries, this proportion reaches more than 3.0%. However, epidemiological reports raise major concern and issues regarding the epigenetic consequences of ART manifested at different stages of pre-, peri- and post-implantation and post-natal development. Hence, the evaluation of the epigenetic status of ART embryo, especially blastocyst, as the final *in vitro* product of ART, would be of principal importance. Therefore, in this study, we assessed the methylation status of *H19/IGF2* DMR in intra cytoplasmic sperm injection-derived blastocysts donated from healthy couples enrolled in ART program for family balancing.

Our results showed that the percentages of overall methylated CpGs and hyper-methylated clones of *H19/IGF2* DMR in blastocysts were 37.85±4.87% and 43.75±5.1%, respectively, while this value for lymphocytes was 49.52±1.86% and 50%. The values obtained for lymphocytes was relatively similar to those reported previously in literature (the published mean methylation indices of 53.3±3.1%[13], 52.07±6.59%[31], 50±3.0%[32] and 49.8%[24]). Likewise, when defined the overall methylation of CpGs of *H19/IGF2* DMR in blastocysts, we observed that the methylation status of the *H19* DMR is lower than the expected methylation level of somatic cells (Fig. 2). This observation, to a certain degree, is in concordance with a previous report from the Lefèvre group[21]. Using genome-wide analysis of DNA methylation of early human blastocyst embryo, Okae *et al*.[33] also reported an average of methylation levels with a median of around 40% for gDMRs, which is close to the value reported in this study and Lefèvre group’s study[21]. It is important to note that Okae and colleagues[33] did not specifically report the methylation level of ICR1. To our knowledge, there is no other study to assess the DMR in high-quality human blastocysts with specific attention to gDMRs or ICR1. Therefore, the values obtained in this study and those reported by others[21,33], which are close to each other, can be considered as reference value for future studies.

In contrast to our results and the aforementioned studies[21,33], Chen *et al*.[27] reported higher degree of DNA methylation (49.4%±9.7% as compared to our value 37.85±4.87%) in human embryos. However, the main difference between our study and Chen *et al.*,

The number of clones is shown in the raw of “Seq. identifier”. Seq, sequence study is that we used high-quality blastocysts donated by healthy couples, while their study was restricted to poor-quality embryos derived during ART procedure from infertile couples. Furthermore, they have not used the result of aberrant methylation or hypo-methylated embryos in this mean value. We believe that if they had included these embryos in their calculation, they might have reached a value close to those of this study and other aforementioned studies. Chen et al.[27] have also confirmed their procedure using leukocytes and concluded the lack of bias in bisulfite PCR. Furthermore, if they included all the embryos in their study, they could achieve a high-degree of standard deviation as compared to the low value obtained in this study, which may be accounted by poor-quality of embryos used in that study. Investigations on mice have also revealed that the methylation level of paternal allele in pre-implantation embryo derived from spontaneously ovulated females is reduced to 77%-79% methylation on paternal allele at H19 ICR as compared to sperm, which is considered to be 100%[34]. This 20% reduction may be due to dynamic changes occurring during normal development. Although such comparison is impossible, the reduced methylation observed here might be the consequence of normal course of development observed in mice.

**Fig. 3:**
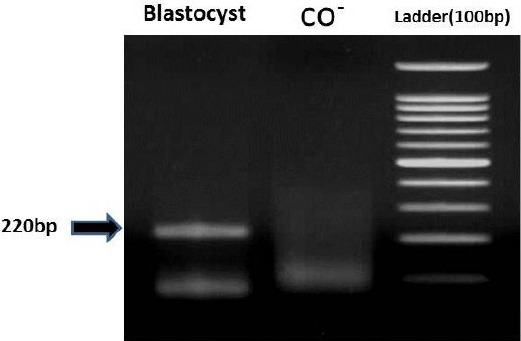
Amplification of bisulfite-converted DNA after nested PCR in blastocysts.

Whether the observed methylation status of the blastocyst is considered as a normal phenotype for these embryos remains to be answered, but if we consider this phenomenon as a divergence from the norm, numerous explanations can be provided that include: 1) paternally-derived altered methylation[35-37], 2) maternally-derived altered methylation[38], 3) reduced maintenance of DNA methylation[29,39], 4) Non-rigid and dynamic methylation statuses of DMR [40-42], 5) reduced or altered methylation due to ART procedure[30,43], and 6) altered methylation due to technical issues. Each of these propositions requires to be evaluated in the context of new experiments.

Since the embryos in this study were derived from healthy volunteer couples with proven fertility among the aforementioned proposition, two possibilities are more likely. Firstly, methylation status of DMR is not rigid and may be dynamic as observed in *in vivo*-derived mouse embryos from non-stimulated cycles[34]. Despite a general consensus that the methylation status of DMRs is persistent to genome-wide demethylation, recent evidence suggests that DMR can be affected at least in part by the genome-wide demethylation during mouse pre-implantation development[42]. In this regard, Tomizawa *et al*.[40] have demonstrated that murine DMRs are not fully protected from the major epigenetic reprogramming events occur during pre-implantation development. Instead, the DMRs appear to be demethylated and show dynamic changes in CpG methylation in blastocysts. Secondly, reduced methylation is due to the ART procedure, which a growing body of researchers has emphasized on this issue[19].

The present study show that the mean methylation status of ICR1 of the human good-quality blastocyst donated by healthy individuals is around 37.85±4.87%. However, since there is no comparison with *in vivo* embryos in human, it is not possible to conclude that the methylation found in the CpG is actually normal or abnormal. Furthermore, ART procedures should be optimized in order to at least reduce some of the risks associated with ART.

## References

[1] Guo H, Zhu P, Yan L, Li R, Hu B, Lian Y, Yan J, Ren X, Lin S, Li J, Jin X, Shi X, Liu P, Wang X, Wang W, Wei Y, Li X, Guo F, Wu X, Fan X, Yong J, Wen L, Xie SX, Tang F, Qiao J (2014). The DNA methylation landscape of human early embryos. Nature.

[2] Barbaux S, Gascoin-Lachambre G, Buffat C, Monnier P, Mondon F, Tonanny MB, Pinard A, Auer J, Bessières B, Barlier A, Jacques S, Simeoni U, Dandolo L, Letourneur F, Jammes H, Vaiman D (2012). A genome-wide approach reveals novel imprinted genes expressed in the human placenta. Epigenetics.

[3] Messerschmidt DM, Knowles BB, Solter D (2014). DNA methylation dynamics during epigenetic reprogramming in the germline and preimplantation embryos. Genes and development.

[4] Morgan HD, Santos F, Green K, Dean W, Reik W (2005). Epigenetic reprogramming in mammals. Human molecular genetics.

[5] Smallwood SA, Tomizawa S, Krueger F, Ruf N, Carli N, Segonds-Pichon A, Sato S, Hata K, Andrews SR, Kelsey G (2011). Dynamic CpG island methylation landscape in oocytes and preimplantation embryos. Nature genetics.

[6] Denomme MM, Mann MR (2012). Genomic imprints as a model for the analysis of epigenetic stability during assisted reproductive technologies. Reproduction.

[7] Barlow DP, Bartolomei MS (2014). Genomic imprinting in mammals. Cold spring harbor perspectives in biology.

[8] Nativio R, Sparago A, Ito Y, Weksberg R, Riccio A, Murrell A (2011). Disruption of genomic neighbourhood at the imprinted IGF2-H19 locus in Beckwith–Wiedemann syndrome and Silver–Russell syndrome. Human molecular genetics.

[9] Murrell A, Heeson S, Reik W (2004). Interaction between differentially methylated regions partitions the imprinted genes Igf2 and H19 into parent-specific chromatin loops. Nature genetics.

[10] Kurukuti S, Tiwari VK, Tavoosidana G, Pugacheva E, Murrell A, Zhao Z, Lobanenkov V, Reik W, Ohlsson R (2006). CTCF binding at the H19 imprinting control region mediates maternally inherited higher-order chromatin conformation to restrict enhancer access to Igf2. Proceedings of the national academy of sciences.

[11] Bartolomei MS (2009). Genomic imprinting:employing and avoiding epigenetic processes. Genes and development.

[12] Cooper WN, Luharia A, Evans GA, Raza H, Haire AC, Grundy R, Bowdin SC, Riccio A, Sebastio G, Bliek J, Schofield PN, Reik W, Macdonald F, Maher ER (2005). Molecular subtypes and phenotypic expression of Beckwith–Wiedemann syndrome. European journal of human genetics.

[13] Gicquel C, Rossignol S, Cabrol S, Houang M, Steunou V, Barbu V, Danton F, Thibaud N, Le Merrer M, Burglen L, Bertrand AM, Netchine I, Le Bouc Y (2005). Epimutation of the telomeric imprinting center region on chromosome 11p15 in Silver-Russell syndrome. Nature genetics.

[14] Netchine I, Rossignol S, Dufourg M-N, Azzi S, Rousseau A, Perin L, Houang M, Steunou V, Esteva B, Thibaud N, Demay MC, Danton F, Petriczko E, Bertrand AM, Heinrichs C, Carel JC, Loeuille GA, Pinto G, Jacquemont ML, Gicquel C, Cabrol S, Le Bouc Y (2007). 11p15 imprinting center region 1 loss of methylation is a common and specific cause of typical Russell-Silver syndrome:clinical scoring system and epigenetic-phenotypic correlations. The Journal of clinical endocrinology and metabolism.

[15] Gaston V, Le Bouc Y, Soupre V, Burglen L, Donadieu J, Oro H, Audry G, Vazquez MP, Gicquel C (2001). Analysis of the methylation status of the KCNQ 1 OT and H 19 genes in leukocyte DNA for the diagnosis and prognosis of Beckwith–Wiedemann syndrome. European journal of human genetics.

[16] Lefebvre L, Viville S, Barton SC, Ishino F, Keverne EB, Surani MA (1998). Abnormal maternal behaviour and growth retardation associated with loss of the imprinted gene Mest. Nature genetics.

[17] Charalambous M, Smith FM, Bennett WR, Crew TE, Mackenzie F, Ward A (2003). Disruption of the imprinted Grb10 gene leads to disproportionate overgrowth by an Igf2-independent mechanism. Proceedings of thenational academy of sciences.

[18] Leighton PA, Ingram RS, Eggenschwiler J, Efstratiadis A, Tilghman SM (1995). Disruption of imprinting caused by deletion of the H19 gene region in mice. Nature.

[19] Hiura H, Okae H, Chiba H, Miyauchi N, Sato F, Sato A, Arima T (2014). Imprinting methylation errors in ART. Reproductive medicine and biology.

[20] Fortier AL, Lopes FL, Darricarrère N, Martel J, Trasler JM (2008). Superovulation alters the expression of imprinted genes in the midgestation mouse placenta. Human molecular genetics.

[21] Ibala-Romdhane S, Al-Khtib M, Khoueiry R, Blachère T, Guérin JF, Lefèvre A (2011). Analysis of H19 methylation in control and abnormal human embryos, sperm and oocytes. European journal of human genetic.

[22] Gardner DK, Weissman A, Howles CM (2012). Textbook of Assisted Reproductive Techniques Fourth Edition:Volume 2:Clinical Perspectives.

[23] Wilton L (2011). Chromosomal mosaicism in day-3 embryos from young, successful art patients as determined by array comparative genomic hybridization (CGH). Fertility and sterility.

[24] Borghol N, Lornage J, Blachère T, Sophie Garret A, Lefèvre A (2006). Epigenetic status of the H19 locus in human oocytes following in vitro maturation. Genomics.

[25] Miller SA1, Dykes DD, Polesky HF (1988). A simple salting out procedure for extracting DNA from human nucleated cells. Nucleic acids research.

[26] Herman JG, Graff JR, Myöhänen S, Nelkin B D, Baylin S B (1996). Methylation-specific PCR:a novel PCR assay for methylation status of CpG islands. Proceedings of the national academy of sciences.

[27] Chen S-L, Shi X-Y, Zheng H-Y, Wu FR, Luo C (2010). Aberrant DNA methylation of imprinted H19 gene in human preimplantation embryos. Fertility and sterility.

[28] Davis TL, Trasler JM, Moss SB, Yanga GJ, Bartolomei MS (1999). Acquisition of the H19 methylation imprint occurs differentially on the parental alleles during spermatogenesis. Genomics.

[29] Cheng K-R, Fu X-W, Zhang R-N, Jia GX1, Hou YP2, Zhu SE (2014). Effect of oocyte vitrification on deoxyribonucleic acid methylation of H19, Peg3, and Snrpn differentially methylated regions in mouse blastocysts. Fertility and sterility.

[30] Khoueiry R, Ibala-Romdhane S, Al-Khtib M, Blachère T, Lornage J, Guérin JF, Lefèvre A (2013). Abnormal methylation of KCNQ1OT1 and differential methylation of H19 imprinting control regions in human ICSI embryos. Zygote.

[31] Schönherr N, Meyer E, Eggermann K, Ranke MB, Wollmann HA, Eggermann T (2006). (Epi) mutations in 11p15 significantly contribute to Silver–Russell syndrome:but are they generally involved in growth retardation?. European journal of medical genetics.

[32] Bliek J, Terhal P, van den Bogaard M-J, Saskia Maas, Ben Hamel, Georgette Salieb-Beugelaar, Marleen Simon, Tom Letteboer, Jasper van der Smagt, Hester Kroes, Marcel Mannens (2006). Hypomethylation of the H19 gene causes not only Silver-Russell syndrome (SRS) but also isolated asymmetry or an SRS-like phenotype. The American journal of human genetics.

[33] Okae H, Chiba H, Hiura H, Hamada H, Sato A, Utsunomiya T, Kikuchi H, Yoshida H, Tanaka A, Suyama M, Arima T (2014). Genome-wide analysis of DNA methylation dynamics during early human development. PLoS genetics.

[34] Market-Velker BA, Zhang L, Magri LS, Bonvissuto AC, Mann MR (2010). Dual effects of superovulation:loss of maternal and paternal imprinted methylation in a dose-dependent manner. human molecular genetics.

[35] Marques CJ, Carvalho F, Sousa M, Barros A (2004). Genomic imprinting in disruptive spermatogenesis. The lancet.

[36] Sato A, Hiura H, Okae H, Miyauchi N, Abe Y, Utsunomiya T, Yaegashi N, Arima T (2011). Assessing loss of imprint methylation in sperm from subfertile men using novel methylation polymerase chain reaction Luminex analysis. Fertility and sterility.

[37] Kobayashi H, Hiura H, John RM, Sato A, Otsu E, Kobayashi N, Suzuki R, Suzuki F, Hayashi C, Utsunomiya T, Yaegashi N, Arima T (2009). DNA methylation errors at imprinted loci after assisted conception originate in the parental sperm. European journal of human genetics.

[38] Paczkowski M, Schoolcraft W, Krisher R (2015). Dysregulation of methylation and expression of imprinted genes in oocytes and reproductive tissues in mice of advanced maternal age. Journal of assisted reproduction and genetics.

[39] Bonakdar E, Edriss M, Bakhtari A, Jafarpour F, Asgari V, Hosseini SM, Boroujeni NS, Hajian M, Rahmani HR, Nasr-Esfahani MH (2015). A physiological, rather than a superovulated, post-implantation environment can attenuate the compromising effect of assisted reproductive techniques on gene expression in developing mice embryos. Molecular reproduction and development.

[40] Tomizawa S-i, Kobayashi H, Watanabe T, Andrews S, Hata K, Kelsey G, Sasaki H (2011). Dynamic stage-specific changes in imprinted differentially methylated regions during early mammalian development and prevalence of non-CpG methylation in oocytes. Development.

[41] O’Doherty AM, Magee DA, O’Shea LC, Forde N, Beltman ME, Mamo S, Fair T (2015). DNA methylation dynamics at imprinted genes during bovine pre-implantation embryo development. BMC developmental biology.

[42] Reik W, Dean W, Walter J (2001). Epigenetic reprogramming in mammalian development. Science.

[43] Sato A, Otsu E, Negishi H, Utsunomiya T, Arima T (2007). Aberrant DNA methylation of imprinted loci in superovulated oocytes. Human reproduction.

